# Mitigation of High Temperatures with *Ascophyllum nodosum* Biostimulants in Papaya (*Carica papaya* L.) Seedlings

**DOI:** 10.3390/plants14030317

**Published:** 2025-01-22

**Authors:** Thayanne Rangel Ferreira, Johnny da Silva Rodrigues, Jean Karlos Barros Galote, Jeane Crasque, Basílio Cerri Neto, Antelmo Ralph Falqueto, Lúcio de Oliveira Arantes, Sara Dousseau Arantes

**Affiliations:** 1 Department of Biological Sciences, Center for Human and Natural Sciences, Federal University of Espírito Santo, Avenida Fernando Ferrari 514, Goiabeiras, Vitória 29075-910, Espírito Santo, Brazilsaradousseau@gmail.com (S.D.A.); 2 Capixaba Institute for Research, Technical Assistance and Rural Extension, BR 101 North, Kilometer 151, Linhares 29915-140, Espírito Santo, Brazil

**Keywords:** bioassay, *Carica papaya* L., gas exchange, plant nutrition

## Abstract

High temperatures can interfere with plant metabolism and physiology, compromising productivity. One tactic to minimize the effects of heatwaves on agriculture is the use of bio-stimulants. This study evaluated two commercial products (Baltiko^®^ and Acadian^®^) containing *Ascophyllum nodosum* in ‘Aliança’ papaya (*Carica papaya* L.) seedlings. Six doses (0, 1, 2, 3, 4, and 8 mL L^−1^) were applied weekly for four weeks at two distinct times, considering moderate and high temperatures. The results indicated distinct effects on gas exchange, seedling development, and nutrient content in leaves and roots. During the moderate temperature period, increasing doses enhanced gas exchange and aerial development, along with increases in potassium and boron levels in the leaves, while root growth decreased. Acadian^®^ provided higher levels of boron in leaves and roots compared to Baltiko^®^. During the period of elevated temperature, increases were observed in leaf area, root dry mass, and leaf content of phosphorus, potassium, sulfur, and zinc, along with potassium in the roots. These increases were primarily attributed to the effects of the applied biostimulants. A lower dose (3 mL L^−1^) is recommended during mild temperatures, while a higher dose (6 mL L^−1^) is suggested for elevated temperatures.

## 1. Introduction

Climate change has significantly increased global temperatures, along with the frequency of extreme events such as severe droughts and more frequent and intense heat waves [1]. These climatic changes pose a challenge to agriculture, especially for papaya (*Carica papaya* L.) cultivation, which can experience up to a 75% reduction in CO_2_ assimilation due to stomatal closure caused by vapor pressure deficit under high temperatures. This restricts CO_2_ entry into RuBisCO [2]. Additionally, stomatal closure increases leaf temperature, negatively impacting photosynthesis by damaging thylakoid membranes, which compromises vegetative growth and production [2]. Therefore, developing new tools to reduce the impact of extreme climatic events on plant productivity has gained considerable attention recently [3].

Plant biostimulants are defined as “biologically derived products designed to enhance plant productivity through unique or emergent properties of their constituents, which go beyond the simple presence of essential nutrients, growth regulators, or known protective compounds” [4]. These products have emerged as effective alternative tools to improve plant nutrition and enhance their ability to tolerate environmental stresses [5]. Among biostimulants derived from seaweed, extracts from *Ascophyllum nodosum* are the most extensively studied [6]. This brown alga is rich in bioactive compounds such as polysaccharides, phenolics, and alginic acids, which modulate physiological and biochemical processes in plants, enhancing their resilience to heat stress [7,8].

Under high temperatures, the use of *A. nodosum* in grapevines has been reported to increase stomatal conductance, transpiration, and leaf thermoregulation, facilitating plant recovery after heat-stress periods [9]. These changes favored vegetative growth, promoting greater resistance to adverse temperature conditions [9]. Moreover, *A. nodosum* extract was also reported to improve the performance of lettuce seedlings under heat stress [8]. Studies with grasses showed that *A. nodosum* application conferred heat resistance, attributed to the presence of cytokinin-like substances and increased potassium (K⁺) uptake by plants [10,11].

*Arabidopsis thaliana* plants treated with *A. nodosum* induced the activation of heat stress-associated genes, including several heat shock protein (HSP) families, which help protect plants from heat-induced damage [12]. Additionally, specific formulations of *A. nodosum*, such as PSI-494, promoted the accumulation of soluble sugars in tomato plants. Furthermore, the application of commercial *A. nodosum* extracts, such as Rygex and Super Fifty, increased the macronutrient N, P, K, Ca, and S contents and micronutrient Mg, Zn, Mn, Fe contents in tomato fruits [13]. Similarly, olive trees (*Olea europaea*) treated with *A. nodosum* exhibited higher uptakes of K and Fe [14].

Despite the proven benefits across various production systems, the efficacy of *A. nodosum* extracts can vary substantially depending on factors such as extraction method, final product formulation, and environmental conditions during application [15,16]. Furthermore, there are still gaps in the literature regarding the effects of these biostimulants on crops such as papaya, particularly on seedling quality, which plays a crucial role in early crop development, directly affecting precocity and productivity [17]. Although the study by [18] indicated improvements in the quality of papaya seedlings with the use of A. nodosum, there is a lack of understanding of the physiological and nutritional effects of this biostimulant. It is worth noting that while not specifically proven for papaya, evidence suggests that environmental factors such as high temperatures can influence plant responses to biostimulants, as observed in studies with other species like grapevine [9].

The optimal temperature range for papaya cultivation is between 21 °C and 33 °C, with 25 °C being ideal [19]. However, major commercial papaya plantations are located between latitudes 23° N and 23° S [2]. In some regions, such as Brazilian semi-arid areas, papaya production occurs outside the optimal temperature range, especially during summer, which may compromise productivity [20,21]. Biostimulants are believed to mitigate the adverse effects of high temperatures on seedling quality.

Thus, this study aimed to evaluate the efficacy of two commercial *A. nodosum*-based products, Baltiko^®^ and Acadian^®^, on the quality of ‘Aliança’ papaya seedlings, considering the effects of increasing doses under high-temperature conditions. The focus was to explore biostimulation in terms of gas exchange, vegetative development, and nutrient uptake. The hypothesis was that using high or low doses of commercial *A. nodosum*-based biostimulants in papaya seedlings grown under high temperatures would promote variable responses in growth, development, and physiology, enabling the identification of an optimal dose for each product.

## 2. Results

### 2.1. First Bioassay

As described in the methodology section, the first bioassay was conducted under moderate temperatures. The climatic data show that the daily maximum temperature ranged from 27 to 33 °C, with a daily average of around 26 °C, and the minimum temperature varied between 19 and 24 °C. The accumulated precipitation was relatively low, totaling 431.1 mm throughout the experiment, with most days having values below 10 mm or no precipitation and only a few isolated peaks above 20 mm.

**Figure 1:**
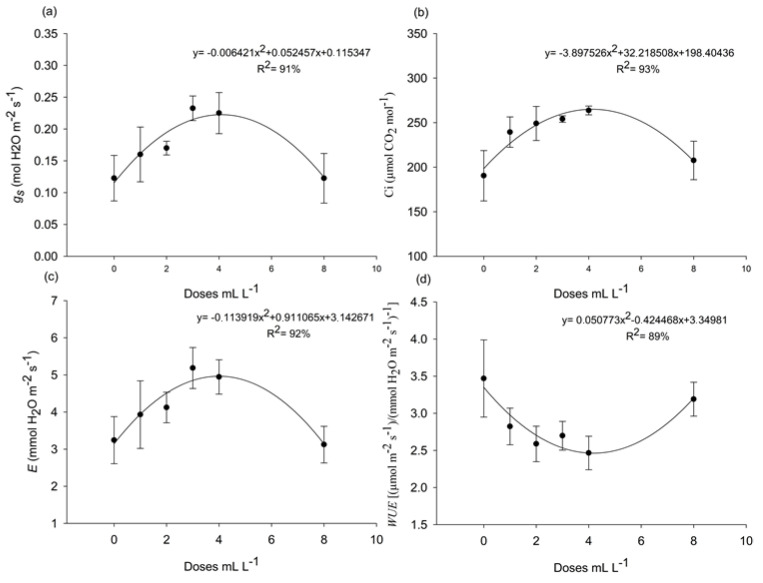
Stomatal conductance (*g_s_*) (**a**), internal carbon dioxide concentration (*C_i_*) (**b**), transpiration (*E*) (**c**), and water use efficiency (*WUE*) (**d**) of ‘Aliança’ papaya seedlings (*Carica papaya* L.) at 48 days after sowing in response to six different doses (0, 1, 2, 3, 4, and 8 mL L^−^^1^) of the commercial product Baltiko^®^ (*A. nodosum*), which was applied weekly for four weeks. The graph considers only the isolated effect of the doses of Baltiko^®^. The bar represents the standard error of four repetitions of 20 plants.

There was no significant interaction between the products and doses for the variables analyzed, with *p*-values for interaction as follows: g_s_ (*p* = 0.0768), C_i_ (*p* = 0.1403), E (*p* = 0.1636), and WUE (*p* = 0.2806). For the product factor, all variables showed *p* > 0.05. However, for the dose factor, only C_i_ showed *p* < 0.05. Nonetheless, the *p*-values of the adjusted model indicated significant effects of A. nodosum doses in the commercial product Baltiko^®^ on g_s_ (*p* = 0.004), C_i_ (*p* = 0.001), E (*p* = 0.009), and WUE (*p* = 0.038). Therefore, the effects of the doses were analyzed independently and fitted to polynomial equations, as illustrated in Figure 1.

*g_s_*, *C_i_*, and *E* showed a quadratic response to the A. nodosum doses, with a maximum value at the dose of 4 mL L^−^^1^ (Figure 1a–c). However, *WUE* exhibited the opposite behavior (Figure 1d). *WUE* decreased at the initial doses, reaching a minimum at 4 mL L^−^^1^, but as the concentration increased, *WUE* showed a growth trend (Figure 1d).

**Figure 2:**
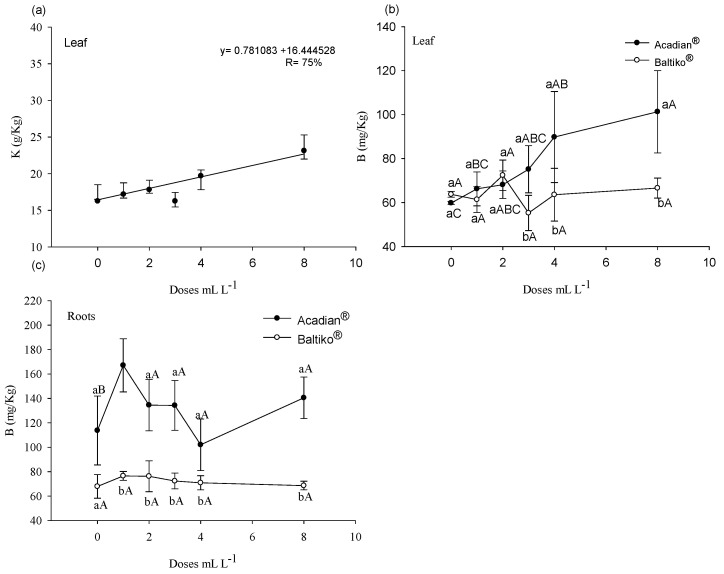
Nutrient levels in leaves and roots of ‘Aliança’ papaya seedlings (*Carica papaya* L.) at 48 days after sowing in response to six different doses (0, 1, 2, 3, 4, and 8 mL L^−^^1^) of *A. nodosum* from the commercial products Baltiko^®^ and Acadian^®^, which were applied weekly for four weeks. Graphs (**a**,**b**) represent potassium (K) and boron (B) in the leaves, while graph (**c**) shows boron (B) in the roots. Vertical bars indicate the standard error of four repetitions of 20 plants. Graphs (**a**,**b**) consider only the isolated effect of the doses, while graph (**c**) represents the interaction between products and doses. Means followed by the same letter do not differ statistically, according to the Tukey test at a 5% probability level. Lowercase letters (vertical) indicate differences between “Products”, and uppercase letters (horizontal) indicate differences between “Doses”.

In leaves, there was no significant interaction between the products and *A. nodosum* doses for potassium (K) contents (*p* = 0.2158). For the product factor, the *p*-value was greater than 0.05 (*p* > 0.05), while for the dose factor, the K content presented a significant *p*-value (*p* < 0.05). In the absence of interaction, the factors were analyzed independently. The effect of *A. nodosum* doses in the commercial products Baltiko^®^ and Acadian^®^ on K content followed a significant linear trend (*p* = 0.001) in the adjusted model, described by a polynomial equation. An increase in K content in leaves was observed as a function of the evaluated doses (Figure 2a), highlighting the positive effect of increasing *A. nodosum* application.

In leaves and roots, a significant interaction was observed between the products and doses for boron (B) content (Figure 2b,c). In leaves, Acadian^®^ showed higher means compared to Baltiko^®^ at doses of 3, 4, and 8 mL L^−^^1^. Analyzing the means of Acadian^®^ doses, the dose of 8 mL L^−^^1^ had the highest mean. On the other hand, comparing the means of Baltiko^®^ doses, no significant differences were found. In roots, Acadian^®^ showed higher means than Baltiko^®^ at all doses, except for the control (Figure 2c). Examining the means of Acadian^®^ doses, all were higher than the control, but no differences were observed among them. Conversely, for Baltiko^®^ doses, no differences were observed.

**Figure 3:**
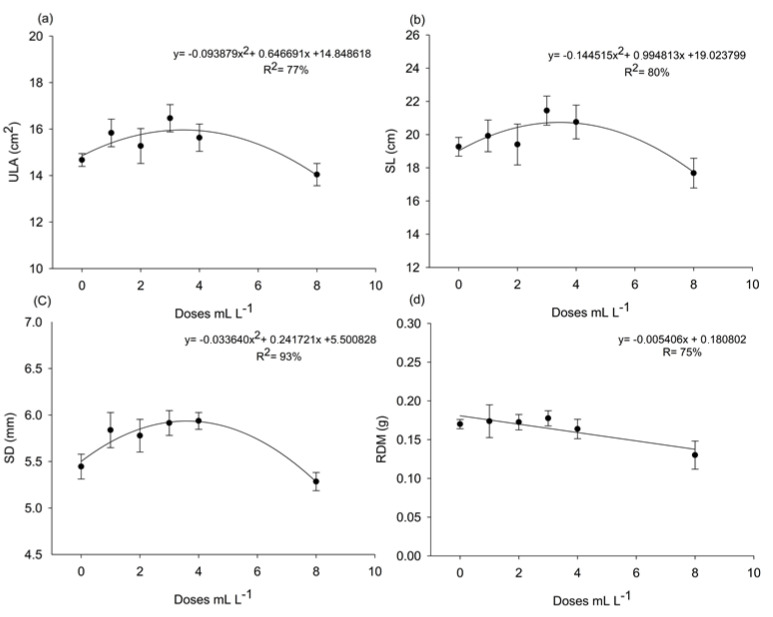
Unit leaf area (**a**), stem length (**b**), stem diameter (**c**), and root dry mass (**d**) of ‘Aliança’ papaya seedlings (*Carica papaya* L.) at 48 days after sowing in response to six different doses (0, 1, 2, 3, 4, and 8 mL L^−1^) of *A. nodosum* from the commercial products Baltiko^®^ and Acadian^®^, which were applied weekly for four weeks. Graphs (**a**–**d**) consider only the isolated effect of the product doses. Vertical bars indicate the standard error of four repetitions of 20 plants.

There was no significant interaction between the factors product and dose for the variables ULA, SL, SD, and DRM, as indicated by the *p*-values for the following interactions: ULA (*p* = 0.5418), SL (*p* = 0.4097), SD (*p* = 0.5369), and DRM (*p* = 0.7953). For the product factor, all variables showed *p* > 0.05. However, for the dose factor, only ULA and SD showed *p* < 0.05. These results indicate that the effects of the factors can be analyzed independently. Polynomial fitting to the data revealed that *A. nodosum* doses had significant effects, with *p*-values of the adjusted model equal to 0.010 for ULA and SL, 0.000 for SD, and 0.028 for DRM. ULA and SL showed quadratic responses to the doses, with maximum estimated values of 3.44 mL L^−^^1^ for both ULA and SL, while SD achieved optimal development with 3.59 mL L^−^^1^ of the extract (Figure 3a–c). In contrast, DRM showed a linear decrease with increasing concentrations, indicating an inverse relationship (Figure 3c).

### 2.2. Second Bioassay

As described in the methodology section, the second bioassay was conducted under high temperatures. The maximum temperature frequently exceeded 35 °C, and the average temperature remained consistently higher compared to the first bioassay. The minimum temperature was also higher than in the first bioassay, with daily average values remaining around 26 °C for most of the period. In addition, the accumulated precipitation was lower, totaling 319.6 mm.

The results indicate that Acadian^®^ exhibited significantly higher means for *g_s_*, *C_i_*, and *E*, regardless of the dose used, compared to Baltiko^®^ (Table 1). Statistical analysis confirmed the absence of significant interaction between the product and dose factors for *g_s_* (*p* = 0.6634), *C_i_* (*p* = 0.5606), and *E* (*p* = 0.3719). On the other hand, the isolated effects of the product factor were highly significant, with *p* = 0.0000 for g_s_, *p* = 0.0009 for *C_i_*, and *p* = 0.0031 for *E*. Regarding the dose factor, all variables showed *p* > 0.05.

No significant interaction was observed between products and doses of *A. nodosum* for the leaf contents of P, K, S, and Zn, as evidenced by the high *p*-values for the interaction between factors P (*p* = 0.7675), K (*p* = 0.6530), S (*p* = 0.6393), and Zn (*p* = 0.5629). For the product factor, the nutrients P, K, S, and Zn presented non-significant *p*-values (*p* > 0.05). However, the dose factor showed statistical significance (*p* < 0.05) for all variables. In the adjusted models, the dose effects were significant, with *p* < 0.05 for P (*p* = 0.001), K (*p* = 0.000), S (*p* = 0.000), and Zn (*p* = 0.002).

In roots, there was also no significant interaction between products and doses for K (*p* = 0.9180) and Zn (*p* = 0.9055). The product factor was significant only for Zn, which showed *p* < 0.05. Meanwhile, the dose factor was significant only for K, with *p* < 0.05. In the adjusted model, dose effects were statistically significant only for K (*p* = 0.002).

**Figure 4:**
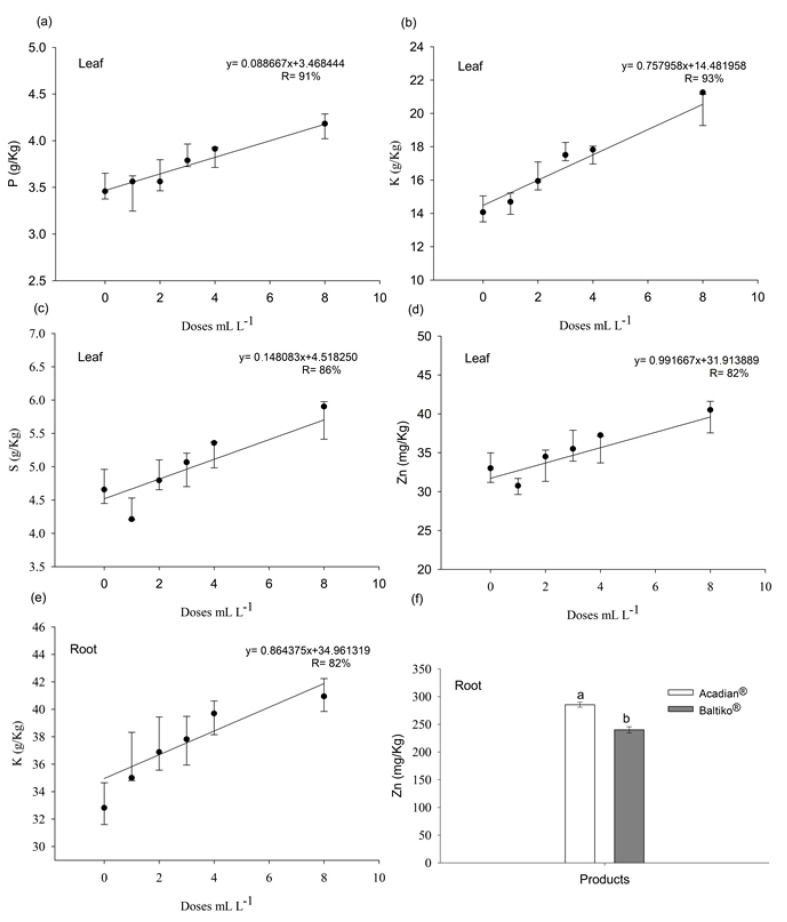
Nutrient levels in leaves and roots of ‘Aliança’ papaya seedlings (*Carica papaya* L.) at 42 days after sowing in response to six different doses (0, 1, 2, 3, 4, and 8 mL L^−^^1^) of *A. nodosum* from the commercial products Baltiko^®^ and Acadian^®^, which were applied weekly for four weeks. Graphs (**a**–**d**) represent potassium (K), boron (B), sulfur (S), and zinc (Zn) in the leaves, while graphs (**e**,**f**) show the same nutrients in the roots. Graphs (**a**–**d**) consider only the isolated effect of the doses, and graphs (**e**,**f**) represent the interaction between products and doses. Vertical bars indicate the standard error of four repetitions of 15 plants. Means followed by the same letter do not differ from each other according to the Tukey test (*p* < 0.05).

The effects of *A. nodosum* doses present in the commercial products Baltiko^®^ and Acadian^®^ on the leaf contents of P, K, S, and Zn, as well as on the root content of K, were described by polynomial equations (Figure 4a–e). A linear relationship was observed between *A. nodosum* doses and the contents of these nutrients, with proportional increases as doses increased (Figure 4a–d). Additionally, the product Acadian^®^ presented higher mean Zn content in roots compared to Baltiko^®^ (Figure 4f).

As in the first bioassay, no significant interaction was observed between product and dose factors for growth variables. The *p*-values for the interaction between the product and doses were as follows: ULA (*p* = 0.4640), SL (*p* = 0.3769), SD (*p* = 0.0006), and DRM (*p* = 0.8838). The *p*-value for the product factor was significant only for SL (*p* = 0.0038) and DRM (*p* = 0.0004), while for the dose factor, it was significant for ULA (*p* = 0.0116), SL (*p* = 0.0028), and SD (*p* = 0.0006).

**Figure 5:**
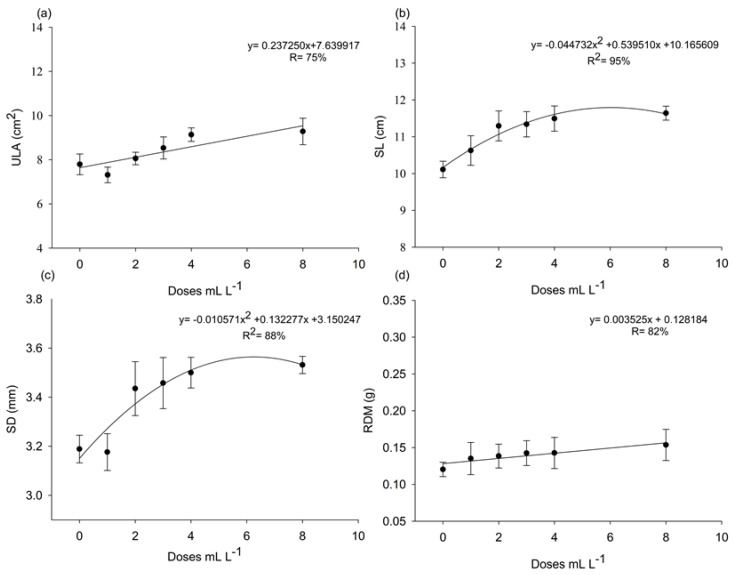
Unit leaf area (**a**), stem length (**b**), stem diameter (**c**), and root dry mass (**d**) of ‘Aliança’ papaya seedlings (*Carica papaya* L.) at 42 days after sowing in response to six different doses (0, 1, 2, 3, 4, and 8 mL L^−^^1^) of A. nodosum from the commercial products Baltiko^®^ and Acadian^®^, which were applied weekly for four weeks. The graph considers only the isolated effect of the product doses. Vertical bars represent the standard error of four repetitions of 15 plants.

The effects of *A. nodosum* doses in the commercial products Baltiko^®^ and Acadian^®^ on ULA, SL, SD, and DRM variables were adequately modeled by polynomial equations (Figure 5). The *p*-values for the adjusted models indicated statistical significance, with *p* = 0.001 for ULA, *p* = 0.014 for SL, *p* = 0.015 for SD, and *p* = 0.044 for DRM, demonstrating that *A. nodosum* doses significantly influenced these variables.

For ULA and DRM, a linear increasing response was observed with higher *A. nodosum* concentrations (Figure 5a,d). In contrast, both SL and SD showed a quadratic response to the doses, with a maximum value at 6 mL L^−^^1^ (Figure 5b,c).

## 3. Discussion

The application of different doses of commercial products containing the bio-stimulant *A. nodosum* in the development of papaya seedlings grown under high temperatures promoted distinct bio-stimulant effects on gas exchange, vegetative growth, and nutrient contents.

It was evident that the 4 mL L^−^^1^ dose of Baltiko^®^ provided positive effects on *g_s_*, *C_i_*, and *E* in the first bioassay (Figure 1). However, in the second bioassay, Acadian^®^ outperformed Baltiko^®^ in these parameters (Table 1). This variation highlights the complexity of interactions between the formulations of seaweed extracts, plants, and the environment. Bioassays often produce varied responses to the same product due to the intricate interactions among plant metabolism, the environment, and the biologically active molecules present in the extracts [22]. Among the active compounds in *A. nodosum* extracts are phenolic compounds [7], organic compounds, and secondary metabolites [8].

Furthermore, the higher dose of 8 mL L^−^^1^ of Baltiko^®^ significantly improved *WUE* (Figure 1d), suggesting that, at higher doses, plants become more efficient in water use. This effect is particularly relevant under the experimental conditions. While temperatures did not reach extreme levels for papaya, they were high enough, as temperatures around 33 °C already significantly increase the water demand of the plant [20]. Additionally, the rise in VPD_air_ during the day, especially in the late afternoon, indicates a low relative humidity atmosphere, which challenges the plant’s water conservation capacity [20]. Hence, the 8 mL L^−^^1^ dose appears to have provided an efficient physiological adjustment, enabling plants to better cope with elevated temperatures, maximizing *WUE*. The improvement in *WUE* may be associated with the increase in K content in leaves (Figure 2a). Potassium (K) is directly related to *WUE* in plants due to its role in stomatal regulation [23]. Studies on grasses have shown that *A. nodosum* application enhanced heat tolerance, attributed to increased K uptake by plants [10,11]. This reinforces the role of K in improving stomatal regulation.

The increase in leaf K in both the first and second bioassays (Figure 2a and Figure 4b) may have positively influenced ULA, SL, and SD, as evidenced in Figure 3 and Figure 5a–c. K’s primary role in photosynthesis is stomatal control, indirectly influencing CO_2_ uptake rates, which, in turn, impacts photosynthesis and enhances plant vegetative development [24]. Additionally, the increase in root K in the second bioassay could have contributed to the rise in DRM (Figure 5d). K stimulates and controls plasma membrane ATPase activity to generate acid stimulation, which triggers cell wall loosening and hydrolase activation, promoting cell growth [25].

The increased uptake of B, P, S, and Zn is amplified by the presence of alginic acid in A. nodosum extracts, benefiting the physical, chemical, and biological properties of the soil [26]. This improved soil water retention capacity, aeration, and capillary action, stimulating plant root systems, microbial activity, and nutrient availability and uptake [27]. Furthermore, analysis of the liquid organic fertilizer (Table 2) and product labels revealed high K levels in both Baltiko^®^ and Acadian^®^, explaining the increase in foliar K with higher doses (Figure 2a and Figure 4b). According to the manufacturer and as referenced by [28], alkaline hydrolysis is the method used to produce A. nodosum extract in these products, justifying the high K content.

The increase in P, S, and Zn in leaves during the second bioassay (Figure 4a–c) positively impacted photosynthetic capacity and shoot growth, as these nutrients are essential for the photochemical phase. P is a structural component of ATP synthesized by ATPases during photochemical reactions [23]. S plays a crucial role in forming iron–sulfur protein complexes involved in the electron transport chain during photosynthesis [29]. Zn is integral to carbonic anhydrase, an enzyme catalyzing CO_2_ and water conversion into bicarbonate and hydrogen ions, essential for guard cell function and stomatal regulation [30,31].

**Figure 6:**
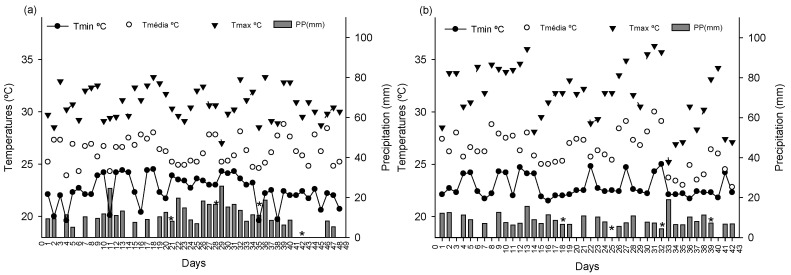
Climatic data from the first (**a**) and second (**b**) bioassays. Minimum temperature (T_min_ °C), maximum temperature (T_max_ °C), average temperature (T_ave_ °C), and precipitation (PPT mm). Asterisks indicate the days of product application.

**Figure 7:**
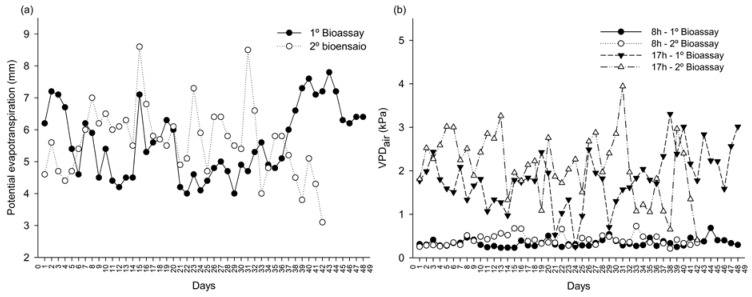
Potential evapotranspiration (**a**) and vapor pressure deficit of the air (**b**) from the first and second bioassay. The first bioassay lasted 42 days, and the second lasted 48 days.

The dose applied in the second bioassay to bio-stimulate ULA, SL, and SD was higher than in the first bioassay, except for DRM. This is likely due to elevated temperatures (Figure 6a,b), increased potential evapotranspiration (Figure 7a), and VPD_air_ (Figure 7b). Maximum temperatures in the second bioassay frequently reached or exceeded 35 °C, accelerating evaporation of the applied product. Even with consistent irrigation, higher temperatures increased evaporation rates, reducing product availability for plants. This necessitated a higher dose to ensure adequate absorption. Moreover, elevated VPD_air_ suggested greater atmospheric water demand, increasing evapotranspiration and product loss, requiring higher doses for efficacy.

Our findings align with [32], demonstrating that heat stress increases potential evapotranspiration and VPD. The mechanisms of seaweed extract action are complex and involve multiple components. There is still limited literature on the bio-stimulant potential of the studied products and the active molecules they contain. Further research is needed to elucidate how these bioactive molecules affect plant growth. Additionally, more studies are required to investigate the use of *A. nodosum* in papaya seedling production and nutrient content.

Our results are consistent with [13], showing that commercial *A. nodosum* extracts (Rygex and Super Fifty) increased macronutrient N, P, K, Ca, and S contents and micronutrient Mg, Zn, Mn, and Fe contents in tomato fruits. Similarly, olive trees treated with *A. nodosum* exhibited higher K and Fe absorption [14]. In tomato and pepper, aerial growth parameters significantly improved with *A. nodosum* treatment compared to controls [27,33].

Beyond leaf area, SD is considered a variable intrinsically related to the seedling’s ability to survive and develop in the field, as taller seedlings with smaller stem diameters are more prone to lodging [34]. Thus, an increase in SD provides a higher chance of survival in the field, particularly during the transplanting stage [34].

As highlighted in the studies by [26] and the review by [8], low concentrations (1:600) of a commercial seaweed extract stimulated tomato root growth, while higher concentrations (1:100) inhibited growth. A similar behavior was observed in the present study for RDM (root dry matter) during the first bioassay (Figure 3d). Reference [35], when evaluating the effects of *A. nodosum* extract application on carrot root growth, also observed that lower doses (0.5 g L^−^^1^ and 0.75 g L^−^^1^) increased root length and diameter, while higher applications (1.0 g L^−^^1^) inhibited growth. Furthermore, when applied at a rate of 0.1% (*v*/*v*), AZAL5, a commercial seaweed extract, improved root growth by stimulating the accumulation of nitrogen and sulfate [36].

In seedling production, having a well-developed root system is an agronomically important trait for any crop, as this attribute influences crop yield, tolerance to abiotic stress, and nutrient uptake and assimilation [37]. According to [38], root growth and development are generally concentrated during the early phenological stages. Thus, a vigorous and well-developed root system during the initial phase is a critical requirement, as it enables the plant to explore the soil more efficiently, ensuring greater production stability.

## 4. Materials and Methods

The bioassays were conducted at the Experimental Farm of the Capixaba Institute for Research, Technical Assistance, and Rural Extension (INCAPER), located at 19°25′00.1″ S and 40°04′35.3″ W in the municipality of Linhares in the northern region of the state of Espírito Santo. The studies were carried out in a greenhouse covered with a black shade cloth with 50% transparency, using micro sprinklers with a flow rate of 7 L per hour (L h^−1^) for three minutes, which were activated every two hours. During this study, climatic data such as minimum, average, and maximum temperature were obtained from the weather station of the National Institute of Meteorology, located in the same region as the experiment, ensuring that the environmental conditions were representative of local commercial cultivation. These data are presented in Figure 6.

Additionally, data on potential evapotranspiration were collected, and from the temperature and humidity, the vapor pressure deficit of the air (VPD_air_ kPa) was calculated (Figure 7). The studies were conducted in Brazil. The first took place from 28 December 2022 to 15 February 2023, a period characterized by moderate temperatures that did not exceed 33 °C. The second was conducted from 23 March to 5 May 2023, during a period of high temperatures, with some days exceeding 35 °C.

The papaya seedlings (*Carica papaya* L.), cultivar Aliança, were propagated from seeds. Three seeds were sown at a depth of 2 cm in 55 cm^3^ capacity tubes containing Tropstrato HT substrate for vegetables. This substrate is composed of pine bark, vermiculite, PG Mix 14.16.18, potassium nitrate, single superphosphate, and peat. Additionally, 1.5 g of Basacote^®^ Mini 3M 16-8-12 (+2) was added per tube. After seedling emergence, thinning was performed to maintain only the most vigorous seedling per tube. Two bioassays were conducted using two commercial products, Baltiko^®^ and Acadian^®^.

According to the manufacturer, Baltiko^®^ consists of *Ascophyllum nodosum* extract, amino acids, humic substances, and water. However, the label does not specify the percentages of seaweed extract and humic substances or the amino acid composition. The product guarantees 5% soluble potassium (63.0 g/L), 2% soluble nitrogen (25.20 g/L), 14% total organic carbon (176.40 g/L), water solubility at 20 °C of 100 g/L, electrical conductivity of 28.50 mS/cm, density of 1.26 kg/L, saline index of 23.50%, pH 7.57, and a maximum recommended solute-to-solvent ratio of 100 g/L. It is described as a fluid suspension.

Acadian^®^ is made from 100% fresh algae and contains 5.3% w/w soluble potassium (61.48 g/L), 6% w/w total organic carbon (69.50 g/L), pH 8.0, density at 20 °C of 1.16 g/mL, saline index of 18%, a maximum solute-to-solvent ratio of 4 mL/L, and 0.5% citric acid as a complexing agent. However, for both products, there is no information on the content of bioactive substances. To complement knowledge about the nutritional effects of the products, samples were sent to the Laboratory of Agronomic, Environmental, and Chemical Solution Preparation Analysis for macro- and micronutrient analysis, with results presented in Table 2.

The experiments were arranged in a randomized block design with a 2 × 6 factorial scheme. The first factor consisted of two commercial sources of *A. nodosum* (Baltiko^®^ and Acadian^®^), and the second factor comprised six doses of each product (0, 1, 2, 3, 4, and 8 mL L^−^^1^). Four blocks were evaluated, with 20 plants per plot in the first study and 15 plants per plot in the second.

After emergence and seedling thinning, six weekly applications of the solutions (0, 1, 2, 3, 4, and 8 mL L^−^^1^) were performed via foliar spraying until runoff occurred and the substrate was saturated. The first application in the first and second bioassays occurred 20 and 12 days after sowing, respectively (Figure 6a,b illustrate the specific days of application). When the seedlings reached the commercial standard of 15–20 cm [39], evaluations of gas exchange and seedling quality were conducted. This occurred 48 days after sowing in the first study and 42 days in the second.

Gas exchange was measured on a mature, fully expanded leaf using an infrared gas analyzer (IRGA 6400 LI-COR, LI-COR Inc., Lincoln, NE, USA). The photosynthetically active radiation (PAR) was set at 1200 μmol, temperature at 30 °C, CO_2_ flow at 300 μmol, and reference CO_2_ at 400 μmol (adapted from Ruas et al., 2020). Two plants per plot were evaluated between 08:00 and 11:00. The evaluated parameters included stomatal conductance (*g_s_*, mol H_2_O m⁻^2^ s^−1^), internal CO_2_ concentration (*C_i_*, µmol CO_2_ mol^−1^), and transpiration rate (*E*, mmol H_2_O m⁻^2^ s^−1^). The instantaneous water-use efficiency (*WUE*, µmol m⁻^2^ s^−1^)/(mmol H_2_O m⁻^2^ s^−1^) was calculated as the ratio of net photosynthesis (P_N_) to *E*.

Shoot development was assessed by leaf area (ULA), stem length (SL), and stem diameter (SD). Root development was evaluated based on root dry mass (DRM). ULA was calculated as the ratio of leaf area to the number of leaves. SL was measured from the collar to the apical bud using a graduated ruler and expressed in cm. SD was measured at the collar region using a precision digital caliper and expressed in millimeters (mm). DRM, expressed in grams, was obtained by weighing the root after drying in a forced-air oven at 65 °C until constant weight.

Dry mass samples of leaves and roots were sent to the Laboratory of Agronomic, Environmental, and Chemical Solution Preparation Analysis for macro- and micronutrient analysis. The analyzed elements included nitrogen (N), phosphorus (P), potassium (K), calcium (Ca), magnesium (Mg), sulfur (S), iron (Fe), zinc (Zn), copper (Cu), manganese (Mn), and boron (B).

Data were subjected to normality tests and analysis of variance (ANOVA). Means were analyzed by polynomial regression, considering a 5% error probability. Means were also compared using Tukey’s test at a 5% probability level. Statistical analyses were performed using SISVAR software, version 4.3 [40].

## 5. Conclusions

The application of *A. nodosum*-based biostimulants showed the potential to improve the quality of ’Aliança’ papaya seedlings by enhancing gas exchange and nutrient uptake. The effectiveness of the commercial products, Baltiko^®^ and Acadian^®^, was dose- and climate-dependent. Baltiko^®^ was particularly effective in improving *WUE*, while Acadian^®^ resulted in higher gas exchange values, irrespective of the dose. Both formulations promoted increased nutrient levels in leaves and roots, with Acadian^®^ showing superior boron uptake. Based on these findings, we recommend adjusting the dose to environmental conditions, with 3 mL L^−1^ for moderate temperatures and 6 mL L^−1^ for high temperatures.

## Figures and Tables

**Table 1:** Stomatal conductance (*g_s_*), internal CO_2_ concentration (*C_i_*), and transpiration (*E*) of ‘Aliança’ papaya seedlings (*Carica papaya* L.) at 42 days after sowing in response to six different doses (0, 1, 2, 3, 4, and 8 mL L^−1^) of *A. nodosum* from the commercial products Baltiko^®^ and Acadian^®^, which were applied weekly for four weeks. The table considers only the isolated effect of the product doses.

Products/Variables	*g_s_* (mol H_2_O m^−2^ s^−1^)	*C_i_* (µmol CO_2_ mol^−1^)	*E* (mmol H_2_O m^−2^ s^−1^)
Acadian^®^	0.25 ^a^	278.02 ^a^	4.23 ^a^
Baltiko^®^	0.17 ^b^	256.8 ^b^	3.72 ^b^

Means followed by the same letter do not differ from each other according to the Tukey test (*p* < 0.05).

**Table 2:** Analysis of macro- and micronutrients of two commercial products, Acadian^®^ and Baltiko^®^, based on *Ascophyllum nodosum* (nitrogen, phosphorus, potassium, calcium, magnesium, sulfur, iron, zinc, copper, manganese, and boron).

Products/Parameters	Acadian^®^			Baltiko^®^		
	Low	Medium	High	Low	Medium	High
Total Nitrogen (%m/m)	1.3					2.2
Total Phosphorus (%m/m)	0.2			0.3		
Total Potassium (%m/m)			5.4			5.7
Total Calcium (%m/m)	0.0995			0.1		
Total Magnesium (%m/m)	0.3			0.03		
Sulfur (%m/m)	0.2					1.9
Iron (%m/m)	0.0017			0.04		
Zinc (ppm)	0.23			0.27		
Copper (ppm)	0.06			0.07		
Manganese (ppm)	0.19			0.47		
Boron (ppm)	0.25			0.42		

## Data Availability

Data are contained within the article.
